# Editorial: Phylogenomic Approaches to Deal With Particularly Challenging Plant Lineages

**DOI:** 10.3389/fpls.2020.591762

**Published:** 2020-11-24

**Authors:** Marcial Escudero, Gonzalo Nieto Feliner, Lisa Pokorny, Daniel Spalink, Juan Viruel

**Affiliations:** ^1^Department of Plant Biology and Ecology, University of Seville, Seville, Spain; ^2^Department of Biodiversity and Conservation, Real Jardín Botánico, CSIC, Madrid, Spain; ^3^Centre for Plant Biotechnology and Genomics (CBGP), Technical University of Madrid (UPM)-National Institute of Agriculture and Food Research and Technology (INIA), Madrid, Spain; ^4^Royal Botanic Gardens, Kew, Richmond, United Kingdom; ^5^Department of Ecology and Conservation Biology, Texas A&M University, College Station, TX, United States

**Keywords:** genotyping-by-sequencing, genome skimming, high-throughput sequencing, phylogenomics, RAD-seq, target capture, Hyb-Seq

Molecular phylogenetics has been revolutionized in the past two decades by the development of increasingly cheap high-throughput sequencing (HTS) technologies. The transition from Sanger to HTS has simultaneously necessitated concerted efforts to develop molecular and computational capacities and technologies, as well as analytical tools to efficiently but rigorously interrogate the resulting massive datasets. Collectively, these approaches are often called “phylogenomics.” Here, we use the term phylogenomics to refer to analyses of HTS data representing large portions of genomes. Indeed, phylogenomics has yielded unprecedented resolution and support to our understanding of the tree of life. At the same time, it has fully corroborated the early finding that the history of individual genes does not mirror the history of lineages and that rapid radiations, both recent and old, are difficult to resolve. The biological processes underlying gene tree discordance remain a challenge to detect and accommodate in most phylogenomic models, and particularly difficult lineages are not immediately clarified by the addition of orders of magnitude more data. Thus, while we have made significant progress in recent years, the remaining road to a resolved tree of life will continue to be challenging.

In this Special Issue, we focused on the use of phylogenomics to shine light into particularly challenging plant lineages. The papers presented herein are intended to illustrate how the application of new analytical approaches and laboratory protocols may tackle the processes underlying biological diversification, thus improving our understanding of lineage relationships through time. From establishing best practices in the development and use of targeted-enrichment bait kits, to formalizing parameter-exploring assembly pipelines, we expect this collection to serve as a useful resource in the planning and execution of successful phylogenomic projects.

Major challenges in phylogenomic approaches ultimately stem from the dynamism of genomes over evolutionary time, but this can be aggravated whenever lineages include hybridization and/or polyploidy in their history (Twyford and Ennos, 2012), since these processes can involve mixing of different phylogenetic signals and an extra amount of change triggered by the so-called genomic shock (McClintock, 1984). Although polyploidy is known to be pervasive in the history of vascular plants (Soltis and Soltis, 2012), detecting whole genome duplication (WGD) events is not trivial due to the suite of genome readjustments that follow every single WGD event and continue over time until a new one occurs (Wendel, 2015). Stai et al. developed a pipeline to identify genome duplications in 14,709 gene families based on evolutionary, phylogenomic, and synteny analyses for the legume family. The study focused on the genus *Cercis* in the legume subfamily Cercidoideae, which they infer as sister to the other legume subfamilies (though see Koenen et al., 2020). They found evidence for a genome duplication (allotetraploidy) in the Cercidoideae subfamily and a set of independent genome duplications in the other legume subfamilies. Due to apomixis, hybridization and polyploidy, *Rubus* (Rosaceae) epitomizes the difficulties in unraveling relationships. Yet, using a target capture dataset comprising plastome and more than nine hundred nuclear loci, from a representative sampling of the rampant variation in ploidy in this genus, Carter et al. managed to untangle the complex phylogenetic relationships in the genus. This included inferring multiple hybridization events among the brambles as well as the biogeographic history of this genus, which involved a North American most recent common ancestor.

The Hawaiian species of the genus *Melicope* (Rutaceae) represent one of the major adaptive radiations of the Hawaiian Islands. Although reduced representation approaches are typically applied to microevolutionary questions, Paetzold et al. relied on RAD-seq to infer phylogenetic relationships, which were poorly resolved using Sanger sequencing. Their results drastically improved resolution of relationships within Hawaiian *Melicope* and, using ABBA-BABA tests, provided evidence for both ancestral and current hybridization events. Donkpegan et al. used a related technique, genotyping-by-sequencing (GBS), to infer diversification dates in African species of the tree genus *Afzelia* (Fabaceae), in which species delimitation and phylogenetic relationships among diploids and tetraploids remained unresolved. Their results suggest that a single biome shift took place from the savannah species, which are diploid, to the rainforest species, which are tetraploid. The implied pattern of an earlier Miocene diversification of the tropical savannah clade compared to the Pliocene diversification of the rainforest clade, is opposite to the one usually found for other groups in the region.

Cost-effective approaches such as genome skimming, which retrieves high-copy number nuclear (e.g., transposons and other repeats) and organellar (i.e., plastome, mitome) regions, have less power for shining light into complex evolutionary scenarios. However, using this technique in *Silene* section *Psammophilae* (Caryophyllaceae), del Valle et al. unveiled clear incongruence between morphology-based taxonomic boundaries and phylogeographic patterns inferred from whole plastomes. This result suggests a history of interspecific hybridization among Iberian populations of the five species that integrate this section, to the exclusion of the Balearic populations of only one (*S. cambessedesii)*. The same technique was used successfully by Moreno-Aguilar et al. to conclude that two enigmatic grass genera, *Megalachne* and *Podophorus* endemic to the Juan Fernandez Pacific archipelago, form a monophyletic group. They also inferred that a long-distance dispersal event gave rise to this group, from South American fescue populations, in the Miocene-Pliocene transition. It is also noticeable that this study is an example of museomics and, remarkably, sampling included a 164-year old type specimen of *Podophorus bromoides*, a species currently considered extinct.

Another challenge to all HTS methods is that handling massive amounts of genomic data generates uncertainty at different stages. Using the Mediterranean genus *Helianthemum* (Cistaceae), Martín-Hernanz et al. established a pipeline to explore the impact of different parameter settings during RAD-seq assembly on genotyping error rates. They found that different parameter configurations produced topologically congruent phylogenies, but also that minimizing error rates results in more reliable branch lengths which affected the accuracy of downstream analyses (i.e., divergence times and diversification rates).

Target sequencing capture approaches are gradually becoming predominant for tackling macroevolutionary questions in non-model organisms. One debated and yet unresolved question concerning these approaches is the selection of loci. It is assumed that specific bait kits designed for the group in question maximize capture. For instance, from a palm-specific enrichment panel targeting 4,184 genomic regions, Loiseau et al. selected 795 phylogenetically informative nuclear markers (PhyloPalm kit) to resolve relationships in palms (Arecaceae). They focused on a widely distributed group of neotropical palms—tribe Geonomateae—and obtained strongly supported topology for this group, whose relationships were previously far from settled. In another study focused on one of the largest tribes in the Asteraceae, Vernonieae, Siniscalchi et al. recovered c. 700 nuclear markers from a kit developed specifically for the family (Mandel et al., 2014) using Hyb-Seq (Weitemier et al., 2014), a HTS approach which combines target enrichment and genome skimming. Although sampling a small percentage of the 1,500 species in the tribe, the authors obtained complete resolution and high support in the phylogeny, substantially improving those in previous studies.

A specific bait kit was also designed for unraveling the neotropical radiation of the spiral ginger (*Costus*, Costaceae) by Valderrama et al. using available genomic resources for *Costus*. They obtained and used 832 loci for phylogenomic analyses—using both concatenation and coalescent-based species trees methods—with which the authors achieved a robust estimation of relationships despite high levels of gene tree conflict. By contrast, some studies rely on phylogenetically broad bait kits, such as the Angiosperms353 kit, which has been carefully designed to capture 353 single-copy nuclear loci across angiosperms, providing useful phylogenetic signal at different phylogenetic depth levels (Johnson et al., 2018). For instance, research in yet another tropical group in this issue uses this Angiosperm353 bait kit and the aforementioned Hyb-Seq approach to investigate the evolutionary history of the Papuasian *Schefflera* (Araliaceae) radiation (Shee et al.). By resolving both deep and shallow phylogenetic relationships, the authors show the efficacy of this universal bait kit, even when sampling herbarium material (including type specimens). They also inferred a sequence of colonization events to explain the present-day distribution of this genus in Papuasia. Concerning the hot question of which bait kit to use in target enrichment approaches, Larridon et al. present an interesting comparison between the Angiosperm353 bait kit and a Cyperaceae-specific kit to unravel the rapid radiation of the C4 *Cyperus* clade. The results are as unexpected as fascinating.

## Author Contributions

All authors listed have made a substantial, direct and intellectual contribution to the work, and approved it for publication.

## Conflict of Interest

The authors declare that the research was conducted in the absence of any commercial or financial relationships that could be construed as a potential conflict of interest.

## References

[B1] Johnson M. G. Pokorny L. Dodsworth S. Botigué L. R. Cowan R. S. Devault A. (2018). A universal probe set for targeted sequencing of 353 nuclear genes from any flowering plant designed using K-medoids clustering. Syst. Biol. 68, 594–606. 10.1093/sysbio/syy086 PMC6568016 30535394

[B2] Koenen E. J. Ojeda D. I. Steeves R. Migliore J. Bakker F. T. Wieringa J. J.. (2020). Large-scale genomic sequence data resolve the deepest divergences in the legume phylogeny and support a near-simultaneous evolutionary origin of all six subfamilies. New Phytol. 225, 1355–1369. 10.1111/nph.16290 31665814 PMC6972672

[B3] Mandel J. R. Dikow R. B. Funk V. A. Masalia R. R. Staton S. E. Kozik A.. (2014). A target enrichment method for gathering phylogenetic information from hundreds of loci: an example from the Compositae. Appl. Plant Sci. 2:1300085. 10.3732/apps.1300085 25202605 PMC4103609

[B4] McClintock B. (1984). The significance of responses of the genome to challenge. Science 226, 792–801. 10.1126/science.15739260 15739260

[B5] Soltis P. S. Soltis D. E. (2012). Polyploidy and Genome Evolution. Berlin: Springer.

[B6] Twyford A. D. Ennos R. A. (2012). Next-generation hybridization and introgression. Heredity 108, 179–189. 10.1038/hdy.2011.68 21897439 PMC3282392

[B7] Weitemier K. Straub S. C. K. Cronn R. C. Fishbein M. Schmickl R. McDonnell A.. (2014). Hyb-Seq: combining target enrichment and genome skimming for plant phylogenomics. Appl. Plant Sci. 2:1400042. 10.3732/apps.1400042 25225629 PMC4162667

[B8] Wendel J. F. (2015). The wondrous cycles of polyploidy in plants. Am. J. Bot. 102, 1753–1756. 10.3732/ajb.1500320 26451037

